# A Prospective Investigation of Body Size, Body Fat Composition and Colorectal Cancer Risk in the UK Biobank

**DOI:** 10.1038/s41598-017-17997-5

**Published:** 2017-12-19

**Authors:** Luisa Saldana Ortega, Kathryn E. Bradbury, Amanda J. Cross, Jessica S. Morris, Marc J. Gunter, Neil Murphy

**Affiliations:** 1 Primary Care Clinical Trials Unit, Nuffield Department of Primary Care Health Sciences, University of Oxford, Oxford, UK; 2 Cancer Epidemiology Unit, Nuffield Department of Population Health, University of Oxford, Oxford, UK; 3 School of Public Health, Imperial College London, London, UK; 4 Section of Nutrition and Metabolism, International Agency for Research on Cancer, Lyon, France

## Abstract

Obesity has been consistently associated with a greater colorectal cancer risk, but this relationship is weaker among women. In the UK Biobank, we investigated the associations between body size (body mass index [BMI], height, waist circumference, and waist-to-hip ratio) and body fat composition (total body fat percentage and trunk fat percentage) measurements with colorectal cancer risk among 472,526 men and women followed for 5.6 years on average. Multivariable hazard ratios (HRs) and 95% confidence intervals (95%CI) for developing colorectal cancer (2,636 incident cases) were estimated using Cox proportional hazards models. Among men, when the highest and lowest fifths were compared, BMI (HR = 1.35, 95%CI: 1.13–1.61; P_trend_ < 0.0001), waist circumference (HR = 1.66, 95%CI: 1.39–1.99; P_trend_ < 0.0001), waist-to-hip ratio (HR = 1.58, 95%CI: 1.31–1.91; P_trend_ < 0.0001), total body fat percentage (HR = 1.27, 95%CI: 1.06–1.53; P_trend_ = 0.002), and trunk fat percentage (HR = 1.31, 95%CI: 1.09–1.58; P_trend_ = 0.002) were associated with greater colorectal cancer risk. For women, only waist-to-hip ratio (HR for highest versus lowest fifth = 1.33, 95%CI: 1.08–1.65; P_trend_ = 0.005) was positively associated with colorectal cancer risk. Greater body size (overall and abdominal adiposity) was positively associated with colorectal cancer development in men. For women, abdominal adiposity, rather than overall body size, was associated with a greater colorectal cancer risk.

## Introduction

A substantial body of evidence has shown that excess adiposity is associated with a greater risk of developing colorectal cancer. A meta-analysis of 30 cohort studies reported that a 5 kg/m^2^ increment in body mass index (BMI) was associated with greater risk of developing colon cancer in both sexes, but that this relationship was weaker among women (relative risk [RR] = 1.12, 95% confidence interval [95%CI]: 1.07–1.18) compared to men (RR = 1.30, 95%CI: 1.25–1.35)^1^. In the same meta-analysis, BMI (per 5 kg/m^2^ increment) was related to rectal cancer risk for men (RR = 1.12, 95%CI: 1.09–1.16) but not women (RR = 1.03, 95%CI: 0.99–1.08)^1^.

Potential reasons for a weaker relationship between adiposity and colorectal cancer among women compared to men are not clear. It has been suggested that hormone replacement therapy (HRT) use, which has been consistently associated with lower risk of developing colorectal cancer^2^, may counteract the detrimental effects of excess adiposity on colorectal tumorigenesis^3^. Support for this hypothesis was provided by the European Prospective Investigation into Cancer and Nutrition (EPIC) study which found that larger waist circumference and waist-to-hip ratio were associated with a greater risk of developing colon cancer among non-HRT users only, with no relationship observed for HRT users^4^. However, inconsistent findings have been reported in other studies which have investigated whether HRT use modified the relationship between obesity and colorectal cancer^5,6^. Additional studies with large numbers of incident cases are required to investigate how adiposity relates to colorectal cancer risk among users and non-users of HRT.

Body fat composition measurements that distinguish between adipose and non-adipose mass can be estimated using bioelectrical impedance. Such technologies, however, have previously been unavailable in large-scale prospective cohort studies so it is uncertain how body fat composition parameters relate to colorectal cancer development.

In the current analysis, we prospectively investigated the associations between colorectal cancer risk and body size (BMI, body weight, height, waist circumference, and waist-to-hip ratio) and body fat composition (total body fat percentage and trunk fat percentage) measurements in the UK Biobank study. The UK Biobank is a large prospective cohort study including over 500,000 participants. The large number of incident colorectal cancer cases provided substantial statistical power to investigate the body size and colorectal cancer relationships according to HRT use, physical activity levels, and across colorectal cancer subsites.

## Materials and Methods

### Study participants

The UK Biobank aims to investigate the genetic, lifestyle, and environmental causes of a range of important diseases^7,8^. Men (n = 229,182) and women (n = 273,474) aged 40 to 69 years who were registered with the NHS between 2006 and 2010 were recruited in 22 study centres across the UK. Ethical approval for the UK Biobank was obtained from the North West Multi-centre Research Ethics Committee (reference number 06/MRE08/65), the National Information Governance Board for Health and Social Care in England and Wales, and the Community Health Index Advisory Group in Scotland. All methods were performed in accordance with the relevant guidelines and regulations of these approvals. At recruitment all participants gave informed consent to participate in UK Biobank and be followed-up, using a signature capture device. During the baseline recruitment visit, participants were asked to complete a touchscreen self-administered questionnaire, which included questions on socio-demographics (including age, education and Townsend deprivation score), health and medical history, lifestyle exposures (including smoking habits, dietary intakes, physical activity, and alcohol consumption), early life exposures, and medication use. Exclusions prior to the onset of analyses included: participants with prevalent cancer at recruitment (n = 27,058) and those with missing body size and composition measurements (n = 3,072). Therefore, our analysis included 472,526 participants (217,700 men and 254,826 women).

### Body size and composition measurements

At the baseline assessment centre visit, trained staff used standard procedures to collect and record the body size and composition measurements. Body weight was measured using the Tanita BC-418MA body composition analyser. Participants were asked to remove their shoes and heavy outer clothing before weighing^9,10^. Height was measured in a barefoot standing position using the Saca 202 device. BMI was calculated by dividing weight in kilograms by the square of height in metres. Waist circumference and hip circumference were measured using the Wessex non-stretchable sprung tape measurement. Waist-to-hip ratio was calculated by dividing waist circumference by hip circumference. The Tanita BC-418MA body composition analyzer (Tanita, Tokyo, Japan) was used to assess bioelectrical impedance measures including total body fat percentage, and trunk fat percentage.

### Colorectal cancer case ascertainment

Cancer cases within the UK Biobank cohort were identified through linkage to national cancer registries. Complete follow-up was available through 30^th^ November 2014 for England and Wales and 31^st^ December 2014 for Scotland. Cancer incidence data were coded using the 10^th^ Revision of the International Classification of Diseases (ICD-10). Proximal colon cancer included those within the caecum, appendix, ascending colon, hepatic flexure, transverse colon, and splenic flexure (C18.0–18.5). Distal colon cancer included those within the descending (C18.6) and sigmoid (C18.7) colon. Overlapping (C18.8) and unspecified (C18.9) lesions of the colon were also included. Cancer of the rectum included cancer occurring at the rectosigmoid junction (C19) and rectum (C20).

### Statistical analysis

Hazard ratios (HRs) and 95% confidence intervals (CIs) were estimated using Cox proportional hazards models. Age was the primary time variable in all models. Time at entry was age at recruitment. Exit time was age at whichever of the following came first: colorectal cancer diagnosis, death, or the last date at which follow-up was considered complete. Models were stratified by age at recruitment in 5-year categories, Townsend deprivation index fifths, and region of the recruitment assessment centre. Possible non-proportionality was assessed using an analysis of Schoenfeld residuals^11^, with no evidence of nonproportionality being detected. The associations between body size and body fat composition measurements with risks of colorectal cancer, colon cancer, proximal colon cancer, distal colon cancer, and rectal cancer were analysed separately for men and women. Heterogeneity of associations across anatomical cancer subsites was assessed by calculating X^2^ statistics.

Participants were grouped into sex-specific fifths of BMI, body weight, height, waist circumference, waist-to-hip ratio, total body fat percentage, and trunk fat percentage. In additional analyses, each body size and composition measurement was modelled on the continuous scales (BMI [per 2.5 kg/m^2^ increase], body weight [per 5 kg increase], height [per 10 cm increase], waist circumference [per 5 cm increase], waist-to-hip ratio [per 0.25 increase], total body fat percentage [per 5% increase], and trunk fat percentage [per 5% increase]), and BMI was also modelled according to predefined categories (<22, 22–<25 [reference group], 25–<30, 30–<35, ≥35 kg/m^2^). The multivariable models were adjusted for physical activity (metabolic equivalent tasks [MET] hours/week; fifths), smoking status and intensity (never; former; current [<15 cigarettes/day]; current [≥15 cigarettes/day]; current [intensity unknown]; unknown), alcohol consumption frequency (never; special occasions only; 1–3 times/month; 1–2 times/week; 3–4 times/week; daily or almost daily; unknown), family history of colorectal cancer (no; yes; unknown), prevalent diabetes (no; yes; unknown), regular aspirin/ibuprofen use (no; yes; unknown), qualifications (none; national exams at age 16 years [CSEs/O-levels/GCSEs or equivalent]; vocational qualifications [NVQ/HND/HNC] or optional national exams at ages 17 to 18 years [A-levels/AS-levels or equivalent]; other professional qualifications; college/university degree; unknown), ever use of HRT (no, yes, unknown), and frequency of red and processed meat consumption (<2 times/week; 2–3 times/week; 3–4 times/week; ≥4 times/week; unknown). Trend tests across body size and composition measurement fifths were calculated by assigning the median value of each category and modelling as continuous terms in Cox regression models.

The body size and colorectal cancer associations were further assessed across subgroups of ever HRT use, menopausal status (pre-menopausal and post-menopausal), prevalent diabetes (no or yes), and physical activity levels (MET hours/week; thirds). Interaction terms (multiplicative scale) between these variables and body size were included in separate models; the statistical significance of the cross-product terms were evaluated using the likelihood ratio test. To evaluate possible reverse causality, cases diagnosed within the first two years of follow-up were excluded in sensitivity analyses. Statistical tests used in the analysis were all two-sided and a P_value_ of <0.05 was considered statistically significant. Analyses were conducted using Stata version 13.1.

## Results

After a median follow-up time of 5.6 years, 2,636 incident cases of colorectal cancer (1,516 were in men and 1,120 were in women) were diagnosed. Of these cases, 1,731 were colon tumours (874 proximal colon, 769 distal colon, and 88 overlapping or unspecified) and 889 were rectal tumours. Compared with those in the lower fifth, men and women in the highest BMI fifth were less physically active, less likely to be never smokers and frequent alcohol consumers, less likely to have attained college education or university degree, and more likely to be regular aspirin/ibuprofen users and more frequent consumers of red and processed meat (Table 1). In addition, ever HRT use was higher for women in the highest BMI fifth compared to those in the lowest fifth (Table 1).

**Table 1:** Characteristics of study participants by fifths of body mass index (kg/m^2^).

**Baseline Characteristic**	**1**	**2**	**3**	**4**	**5**
**Men**					
**Body mass index (kg/m** ^**2**^ **)**	**<24.5**	**24.5**–<**26.4**	**26.4**–<**28.3**	**28.3**–<**30.8**	**≥30.8**
**Colorectal cancer cases, n**	242	254	309	344	367
**Age at recruitment (years)** ^**†**^	55.9 (8.4)	56.6 (8.3)	56.7 (8.2)	56.8 (8.1)	56.7 (8.0)
**Height (cm)** ^**†**^	176.2 (7.0)	175.9 (6.8)	175.6 (6.8)	175.4 (6.8)	175.1 (6.8)
**Waist circumference (cm)** ^**†**^	84.7 (6.1)	91.3 (5.3)	95.8 (5.4)	100.9 (5.7)	111.9 (9.7)
**Waist-to-hip ratio** ^**†**^	0.88 (0.1)	0.91 (0.1)	0.93 (0.1)	0.96 (0.1)	0.99 (0.1)
**Body fat (%)** ^**†**^	18.9 (4.4)	22.8 (3.7)	25.1 (3.4)	27.5 (3.3)	32.0 (4.0)
**Trunk fat (%)** ^**†**^	20.4 (5.9)	25.0 (4.6)	27.5 (4.1)	30.2 (3.8)	34.9 (4.3)
**Physical activity (MET hour/week)** ^**†**^	43.5 (59.4)	42.1 (57.2)	41.4 (57.3)	40.6 (57.0)	36.5 (55.2)
**Smoking status (%)**					
Never	55.1	52.1	49.1	45.7	42.7
Current	16.1	12.2	11.6	11.6	11.4
**Alcohol consumption (%)**					
Never	6.9	5.6	5.6	5.9	7.3
Daily or almost daily	27.7	27.1	26.4	24.7	20.5
**Qualifications (%)**					
College/university degree	42.9	38.0	33.8	29.1	24.4
**Family history of colorectal cancer (%)**					
Yes	10.2	10.9	11.1	11.4	11.7
**Prevalent diabetes**					
Yes	2.2	3.3	4.4	6.9	14.1
**Regular aspirin/ibuprofen use (%)**					
Yes	21.1	24.8	27.6	31.3	37.3
**Red and processed meat (%)**					
<1 occasion per week	11.3	8.0	6.3	5.7	4.7
≥3 occasions per week	21.5	23.0	24.8	26.9	30.2
**Women**					
**Body mass index (kg/m** ^**2**^ **)**	**<22.9**	**22.9**–<**25.0**	**25.0**–<**27.3**	**27.3**–<**30.8**	**≥30.8**
**Colorectal cancer cases, n**	180	229	259	232	220
**Age at recruitment (years)** ^**†**^	54.5 (8.2)	55.9 (8.0)	56.7 (7.9)	57.1 (7.9)	56.5 (7.8)
**Height (cm)** ^**†**^	163.7 (6.3)	163.0 (6.2)	162.4 (6.2)	161.8 (6.3)	161.3 (6.3)
**Waist circumference (cm)** ^**†**^	71.6 (5.5)	77.7 (5.6)	82.9 (6.1)	89.2 (6.6)	101.8 (10.3)
**Waist-to-hip ratio** ^**†**^	0.77 (0.1)	0.79 (0.1)	0.82 (0.1)	0.84 (0.1)	0.87 (0.1)
**Body fat (%)** ^**†**^	28.0 (4.6)	33.2 (3.6)	36.6 (3.3)	39.9 (3.1)	45.1 (3.8)
**Trunk fat (%)** ^**†**^	25.0 (5.9)	30.7 (4.8)	34.3 (4.6)	37.8 (4.5)	42.6 (5.0)
**Physical activity (MET hour/week)** ^**†**^	37.1 (44.2)	36.0 (42.4)	33.9 (41.4)	31.8 (40.5)	27.9 (38.7)
**Smoking status (%)**					
Never	62.4	60.2	59.2	58.1	58.0
Current	10.2	8.7	8.7	8.7	8.3
**Alcohol consumption (%)**					
Never	8.3	7.2	8.1	10.0	13.6
Daily or almost daily	20.7	19.0	16.8	14.5	9.3
**Qualifications (%)**					
College/university degree	41.1	34.7	29.8	26.6	23.4
**Family history of colorectal cancer (%)**					
Yes	10.0	10.4	10.9	10.7	10.8
**Prevalent diabetes**					
Yes	0.9	1.2	1.9	3.6	9.0
**Regular aspirin/ibuprofen use (%)**					
Yes	20.0	22.8	24.7	27.6	32.3
**Red and processed meat (%)**					
<1 occasion per week	18.3	13.0	11.7	10.2	9.2
≥3 occasions per week	15.4	17.9	19.5	21.2	22.7
**Ever use of hormone replacement therapy (%)**					
Yes	31.4	37.0	39.9	41.4	38.6

^†^Mean and standard deviation

Abbreviation: MET, metabolic equivalent tasks.

### Body size and colorectal cancer risk

Among men, higher BMI was associated with elevated colorectal cancer risk in the multivariable models (HR for highest versus lowest fifth = 1.35, 95%CI: 1.13–1.61; P_trend_ < 0.0001) (Table 2). A similar positive relationship was observed when pre-defined BMI categories were used (HR for BMI ≥35 kg/m^2^ versus 22–<24.9 kg/m^2^ = 1.37, 95%CI: 1.07–1.76; P_trend_ < 0.0001) (data not shown). The positive relationship between BMI and colorectal cancer risk was most apparent for colon cancer versus rectal cancer (P_heterogeneity_ = 0.17), and for proximal colon versus distal colon cancer (P_heterogeneity_ = 0.14), but these differences did not reach statistical significance (Table 2). For women, BMI was not associated with colorectal cancer risk (HR for highest versus lowest fifth = 1.11, 95%CI: 0.89–1.38; P_trend_ = 0.81) (Table 3). Similar relationships were found when pre-defined BMI categories were used (HR for BMI ≥35 kg/m^2^ versus 22–<24.9 kg/m^2^ = 1.03, 95%CI: 0.79–1.34; P_trend_ = 0.43) (data not shown), and across anatomical subsites (colon versus rectal P_heterogeneity_ = 0.30; proximal colon versus distal colon P_heterogeneity_ = 0.17) (Table 3).

**Table 2:** Hazard Ratios (HR) and 95% Confidence Intervals (CI) for colorectal cancer among men by fifths of body size and composition measurements.

	**Colorectal cancer**			**Colon cancer**		**Proximal colon cancer**		**Distal colon cancer**		**Rectal cancer**	
	**N cases**	**Basic**	**Multivariable**	**N cases**	**Multivariable**	**N cases**	**Multivariable**	**N cases**	**Multivariable**	**N cases**	**Multivariable**
							**HR (95%CI)**		**HR (95%CI)**		**HR (95%CI)**
		**HR (95%CI)**	**HR (95%CI)**		**HR (95%CI)**						
**Body mass index, kg/m** ^**2**^											
<24.5	242	1 (Referent)	1 (Referent)	133	1 (Referent)	69	1 (Referent)	60	1 (Referent)	106	1 (Referent)
24.5–<26.4	254	0.99 (0.83–1.18)	0.95 (0.79–1.14)	152	1.01 (0.79–1.28)	71	0.94 (0.66–1.34)	76	1.06 (0.75–1.50)	101	0.91 (0.68–1.20)
26.4–<28.3	309	1.20 (1.01–1.42)	1.15 (0.96–1.37)	189	1.23 (0.98–1.56)	90	1.23 (0.88–1.72)	89	1.15 (0.82–1.62)	117	1.05 (0.79–1.38)
28.3–<30.8	344	1.33 (1.12–1.56)	1.27 (1.07–1.51)	213	1.36 (1.08–1.72)	114	1.59 (1.15–2.19)	94	1.15 (0.81–1.62)	131	1.19 (0.91–1.56)
≥30.8	367	1.44 (1.22–1.69)	1.35 (1.13–1.61)	233	1.50 (1.19–1.90)	113	1.52 (1.09–2.13)	106	1.34 (0.95–1.88)	133	1.17 (0.88–1.55)
*P*_*trend*_		<*0.0001*	<*0.0001*		<*0.0001*		*0.001*		*0.08*		*0.08*
Per 2.5 kg/m^2^ increase		1.08 (1.05–1.11)	1.07 (1.04–1.11)		1.09 (1.05–1.14)		1.12 (1.06–1.19)		1.05 (0.99–1.12)		1.04 (0.99–1.10)
**Weight, kg**											
<74.3	249	1 (Referent)	1 (Referent)	135	1 (Referent)	69	1 (Referent)	62	1 (Referent)	111	1 (Referent)
74.3–<81.1	299	1.20 (1.02–1.42)	1.22 (1.02–1.46)	184	1.35 (1.06–1.71)	87	1.26 (0.89–1.78)	90	1.41 (1.00–1.98)	115	1.09 (0.83–1.43)
81.1–<87.6	296	1.23 (1.04–1.46)	1.24 (1.03–1.49)	181	1.32 (1.04–1.69)	89	1.36 (0.96–1.92)	86	1.26 (0.88–1.80)	113	1.14 (0.86–1.50)
87.6–<96.4	324	1.38 (1.17–1.63)	1.40 (1.17–1.69)	196	1.53 (1.19–1.95)	102	1.66 (1.18–2.34)	85	1.33 (0.93–1.92)	124	1.25 (0.94–1.66)
≥96.4	348	1.58 (1.34–1.86)	1.57 (1.29–1.91)	224	1.79 (1.38–2.30)	110	1.82 (1.27–2.62)	102	1.62 (1.11–2.36)	125	1.33 (0.98–1.80)
*P*_*trend*_		<*0.0001*	<*0.0001*		<*0.0001*		<*0.0001*		*0.03*		*0.05*
Per 5 kg increase		1.05 (1.03–1.06)	1.04 (1.02–1.07)		1.06 (1.03–1.09)		1.08 (1.04–1.12)		1.03 (0.99–1.07)		1.02 (0.99–1.06)
**Height, cm**											
<170	404	1 (Referent)	1 (Referent)	233	1 (Referent)	113	1 (Referent)	111	1 (Referent)	168	1 (Referent)
170–<174	300	0.85 (0.73–0.99)	0.89 (0.76–1.04)	177	0.94 (0.76–1.15)	92	0.96 (0.72–1.29)	77	0.91 (0.67–1.24)	119	0.81 (0.63–1.04)
174–<177	279	1.02 (0.88–1.19)	1.07 (0.91–1.25)	177	1.20 (0.97–1.48)	88	1.21 (0.90–1.63)	86	1.24 (0.92–1.68)	101	0.90 (0.70–1.17)
177–<181	278	0.97 (0.83–1.13)	1.00 (0.85–1.17)	180	1.14 (0.92–1.40)	87	1.11 (0.82–1.50)	85	1.17 (0.86–1.59)	101	0.84 (0.65–1.09)
≥181	255	1.01 (0.86–1.18)	1.01 (0.85–1.19)	153	1.07 (0.86–1.34)	77	1.16 (0.84–1.58)	66	0.94 (0.67–1.32)	99	0.91 (0.70–1.19)
*P*_*trend*_		*0.71*	*0.67*		*0.22*		*0.24*		*0.74*		*0.44*
Per 10 cm increase		1.04 (0.97–1.13)	1.05 (0.96–1.13)		1.09 (0.98–1.22)		1.15 (0.99–1.33)		1.03 (0.88–1.20)		0.97 (0.85–1.11)
**Waist circumference, cm**											
<88	221	1 (Referent)	1 (Referent)	122	1 (Referent)	58	1 (Referent)	62	1 (Referent)	96	1 (Referent)
88–<93	246	1.29 (1.07–1.55)	1.26 (1.04–1.52)	138	1.32 (1.02–1.70)	66	1.37 (0.95–1.99)	64	1.17 (0.81–1.68)	106	1.21 (0.91–1.62)
93–<99	352	1.39 (1.17–1.64)	1.32 (1.11–1.58)	214	1.41 (1.11–1.78)	112	1.64 (1.17–2.30)	94	1.15 (0.82–1.62)	138	1.26 (0.96–1.65)
99–<105	289	1.46 (1.22–1.74)	1.35 (1.12–1.62)	179	1.46 (1.14–1.88)	87	1.59 (1.11–2.28)	88	1.32 (0.93–1.88)	108	1.21 (0.90–1.62)
≥105	408	1.75 (1.48–2.06)	1.66 (1.39–1.99)	267	1.89 (1.49–2.40)	134	2.16 (1.53–3.04)	117	1.46 (1.03–2.06)	140	1.40 (1.06–1.86)
*P*_*trend*_		<*0.0001*	<*0.0001*		*<0.0001*		*<0.0001*		*0.02*		*0.04*
Per 5 cm increase		1.08 (1.05–1.10)	1.07 (1.04–1.10)		1.08 (1.05–1.12)		1.10 (1.05–1.15)		1.06 (1.01–1.11)		1.05 (1.01–1.09)
**Waist-to-hip ratio**											
<0.88	188	1 (Referent)	1 (Referent)	108	1 (Referent)	46	1 (Referent)	58	1 (Referent)	77	1 (Referent)
0.88–<0.92	228	1.10 (0.91–1.34)	1.11 (0.91–1.36)	132	1.14 (0.87–1.49)	70	1.48 (0.99–2.20)	58	0.92 (0.63–1.34)	93	1.08 (0.79–1.47)
0.92–<0.95	319	1.41 (1.18–1.69)	1.36 (1.12–1.64)	195	1.45 (1.13–1.86)	106	2.03 (1.40–2.94)	80	1.02 (0.71–1.46)	127	1.32 (0.99–1.77)
0.95–<0.99	365	1.56 (1.31–1.86)	1.47 (1.22–1.77)	218	1.52 (1.19–1.95)	110	1.98 (1.36–2.87)	97	1.15 (0.81–1.63)	145	1.44 (1.08–1.92)
≥0.99	416	1.70 (1.43–2.02)	1.58 (1.31–1.91)	267	1.73 (1.35–2.21)	125	2.04 (1.40–2.98)	132	1.46 (1.03–2.05)	146	1.42 (1.05–1.91)
*P*_*trend*_		<*0.0001*	<*0.0001*		<*0.0001*		<*0.0001*		*0.006*		*0.005*
Per 0.25 increase		2.00 (1.65–2.42)	1.82 (1.47–2.26)		1.95 (1.48–2.56)		2.04 (1.40–2.98)		1.76 (1.16–2.66)		1.63 (1.16–2.30)
**Total body fat %**											
<20.6	218	1 (Referent)	1 (Referent)	123	1 (Referent)	61	1 (Referent)	60	1 (Referent)	91	1 (Referent)
20.6–<24.0	241	1.01 (0.84–1.21)	1.01 (0.84–1.23)	141	1.00 (0.78–1.29)	76	1.16 (0.80–1.65)	59	0.80 (0.55–1.17)	98	1.05 (0.78–1.41)
24.0–<26.8	296	1.15 (0.96–1.37)	1.14 (0.95–1.37)	181	1.18 (0.92–1.50)	91	1.26 (0.89–1.78)	81	0.99 (0.70–1.41)	112	1.10 (0.83–1.48)
26.8–<30.0	334	1.25 (1.05–1.49)	1.22 (1.02–1.47)	198	1.24 (0.97–1.57)	95	1.26 (0.89–1.78)	94	1.12 (0.80–1.58)	136	1.25 (0.94–1.66)
≥30.0	394	1.36 (1.15–1.61)	1.27 (1.06–1.53)	258	1.36 (1.07–1.72)	124	1.43 (1.01–2.01)	123	1.17 (0.83–1.65)	138	1.18 (0.88–1.58)
*P*_*trend*_		*<0.0001*	*0.002*		*0.002*		*0.04*		*0.11*		*0.15*
Per 5% increase		1.11 (1.06–1.16)	1.08 (1.03–1.14)		1.12 (1.05–1.19)		1.11 (1.01–1.21)		1.10 (0.99–1.22)		1.05 (0.97–1.14)
**Trunk fat %**											
<22.4	219	1 (Referent)	1 (Referent)	117	1 (Referent)	60	1 (Referent)	55	1 (Referent)	98	1 (Referent)
22.4–<26.4	247	1.07 (0.90–1.29)	1.10 (0.91–1.33)	149	1.18 (0.92–1.52)	82	1.35 (0.95–1.93)	63	0.99 (0.68–1.45)	95	1.01 (0.75–1.35)
26.4–<29.6	293	1.16 (0.97–1.38)	1.17 (0.97–1.41)	174	1.25 (0.98–1.60)	85	1.28 (0.90–1.82)	79	1.10 (0.76–1.58)	118	1.11 (0.84–1.47)
29.6–<33.1	328	1.25 (1.05–1.48)	1.23 (1.03–1.48)	202	1.37 (1.08–1.75)	97	1.35 (0.95–1.91)	94	1.27 (0.89–1.80)	125	1.09 (0.82–1.45)
≥33.1	399	1.39 (1.17–1.64)	1.31 (1.09–1.58)	259	1.50 (1.17–1.91)	123	1.48 (1.05–2.10)	126	1.38 (0.98–1.96)	140	1.13 (0.85–1.51)
*P*_*trend*_		<*0.0001*	*0.002*		<*0.0001*		*0.04*		*0.02*		*0.33*
Per 5% increase		1.09 (1.05–1.14)	1.07 (1.02–1.12)		1.10 (1.04–1.17)		1.10 (1.01–1.19)		1.07 (0.99–1.17)		1.04 (0.97–1.12)

Basic model – Cox regression stratified by age (5-year categories), Townsend deprivation index fifths, and region of the recruitment assessment centre. Multivariable model – Cox regression using physical activity (MET hours/week; fifths), smoking status and intensity (never; former; current –<15 cigarettes/day; current –≥15 cigarettes/day; current – intensity unknown; unknown), alcohol consumption frequency (never; special occasions only; 1–3 times/month; 1–2 times/week; 3–4 times/week; daily or almost daily; unknown), family history of colorectal cancer (no; yes; unknown), prevalent diabetes (no; yes; unknown), regular aspirin/ibuprofen use (no; yes; unknown), qualifications (none; CSEs/O-levels/GCSEs or equivalent; NVQ/HND/HNC/A-levels/AS-levels or equivalent; other professional qualifications; college/university degree; unknown), frequency of red and processed meat consumption (<2 times/week; 2–3 times/week; 3–4 times/week; ≥4 times/week; unknown), and stratified by age (5-year categories), Townsend deprivation index fifths, and region of the recruitment assessment centre.

**Table 3:** Hazard Ratios (HR) and 95% Confidence Intervals (CI) for colorectal cancer among women by fifths of body size and composition measurements.

	**Colorectal cancer**			**Colon cancer**		**Proximal colon cancer**		**Distal colon cancer**		**Rectal cancer**	
	**N cases**	**Basic**	**Multivariable**	**N cases**	**Multivariable**	**N cases**	**Multivariable**	**N cases**	**Multivariable**	**N cases**	**Multivariable**
		**HR (95%CI)**	**HR (95%CI)**		**HR (95%CI)**		**HR (95%CI)**		**HR (95%CI)**		**HR (95%CI)**
**Body mass index, kg/m** ^**2**^											
<22.9	180	1 (Referent)	1 (Referent)	128	1 (Referent)	64	1 (Referent)	51	1 (Referent)	51	1 (Referent)
22.9–<25.0	229	1.18 (0.97–1.43)	1.19 (0.97–1.46)	159	1.17 (0.91–1.50)	76	1.13 (0.80–1.60)	72	1.28 (0.88–1.87)	69	1.26 (0.86–1.86)
25.0–<27.3	259	1.27 (1.05–1.54)	1.30 (1.06–1.59)	184	1.32 (1.03–1.67)	98	1.34 (0.96–1.88)	78	1.39 (0.96–2.02)	76	1.34 (0.92–1.96)
27.3–<30.8	232	1.08 (0.89–1.32)	1.13 (0.92–1.40)	170	1.17 (0.91–1.49)	93	1.26 (0.89–1.78)	71	1.18 (0.80–1.73)	58	1.05 (0.70–1.58)
≥30.8	220	1.10 (0.90–1.34)	1.11 (0.89–1.38)	170	1.21 (0.93–1.57)	86	1.25 (0.87–1.80)	72	1.20 (0.80–1.78)	47	0.88 (0.56–1.36)
*P*_*trend*_		*0.94*	*0.81*		*0.31*		*0.27*		*0.74*		*0.24*
Per 2.5 kg/m^2^ increase		1.01 (0.98–1.04)	1.01 (0.98–1.04)		1.02 (0.98–1.06)		1.05 (0.99–1.11)		0.99 (0.93–1.05)		0.98 (0.91–1.04)
**Weight, kg**											
<60.1	192	1 (Referent)	1 (Referent)	134	1 (Referent)	65	1 (Referent)	56	1 (Referent)	58	1 (Referent)
60.1–<66.1	234	1.22 (1.01–1.48)	1.19 (0.97–1.46)	165	1.25 (0.99–1.60)	91	1.42 (1.02–2.00)	65	1.14 (0.78–1.65)	70	1.12 (0.77–1.63)
66.1–<72.3	222	1.14 (0.94–1.38)	1.10 (0.89–1.36)	164	1.19 (0.93–1.52)	77	1.16 (0.81–1.65)	77	1.30 (0.90–1.88)	55	0.91 (0.61–1.35)
72.3–<81.4	248	1.26 (1.04–1.52)	1.19 (0.97–1.47)	179	1.26 (0.98–1.62)	96	1.42 (1.00–2.01)	75	1.21 (0.83–1.77)	67	1.07 (0.72–1.57)
≥81.4	224	1.21 (0.99–1.46)	1.11 (0.89–1.38)	169	1.26 (0.97–1.64)	88	1.42 (0.98–2.06)	71	1.17 (0.79–1.75)	51	0.81 (0.52–1.24)
*P*_*trend*_		*0.10*	*0.56*		*0.17*		*0.13*		*0.53*		*0.27*
Per 5 kg increase		1.02 (0.99–1.04)	1.01 (0.98–1.03)		1.02 (0.99–1.05)		1.04 (0.99–1.08)		0.99 (0.95–1.04)		0.98 (0.93–1.03)
**Height, cm**											
<157	252	1 (Referent)	1 (Referent)	189	1 (Referent)	97	1 (Referent)	80	1 (Referent)	64	1 (Referent)
157–<161	256	1.04 (0.87–1.24)	1.01 (0.84–1.22)	187	1.01 (0.82–1.26)	101	1.11 (0.82–1.50)	80	0.97 (0.70–1.35)	67	0.97 (0.68–1.40)
161–<164	207	1.06 (0.88–1.27)	1.02 (0.84–1.24)	142	0.94 (0.74–1.18)	70	1.02 (0.74–1.41)	59	0.82 (0.57–1.18)	62	1.19 (0.83–1.72)
164–<168	229	1.16 (0.97–1.39)	1.07 (0.88–1.30)	167	1.10 (0.87–1.37)	83	1.12 (0.81–1.54)	72	1.08 (0.77–1.52)	61	0.98 (0.67–1.43)
≥168	176	1.21 (0.99–1.47)	1.11 (0.90–1.37)	126	1.08 (0.84–1.38)	66	1.24 (0.88–1.76)	53	0.97 (0.67–1.42)	47	1.08 (0.72–1.62)
*P*_*trend*_		*0.03*	*0.30*		*0.45*		*0.27*		*0.95*		*0.72*
Per 10 cm increase		1.14 (1.04–1.26)	1.08 (0.98–1.20)		1.06 (0.94–1.20)		1.12 (0.94–1.33)		1.01 (0.84–1.22)		1.09 (0.89–1.33)
**Waist circumference, cm**											
<74	189	1 (Referent)	1 (Referent)	133	1 (Referent)	61	1 (Referent)	56	1 (Referent)	61	1 (Referent)
74–<80	227	1.22 (1.01–1.48)	1.22 (0.99–1.50)	158	1.22 (0.96–1.56)	77	1.33 (0.94–1.89)	67	1.20 (0.83–1.74)	77	1.31 (0.89–1.91)
80–<86	226	1.21 (0.99–1.46)	1.20 (0.98–1.47)	167	1.26 (0.99–1.61)	88	1.38 (0.97–1.95)	73	1.31 (0.91–1.89)	88	1.22 (0.83–1.80)
86–<95	242	1.14 (0.94–1.38)	1.15 (0.94–1.41)	180	1.23 (0.97–1.56)	100	1.48 (1.05–2.08)	76	1.20 (0.83–1.74)	100	1.04 (0.70–1.56)
≥95	236	1.26 (1.03–1.53)	1.22 (0.99–1.52)	173	1.26 (0.97–1.62)	91	1.49 (1.04–2.14)	72	1.16 (0.78–1.71)	91	1.20 (0.79–1.81)
*P*_*trend*_		*0.09*	*0.19*		*0.15*		*0.04*		*0.63*		*0.78*
Per 5 cm increase		1.03 (1.01–1.05)	1.03 (1.00–1.06)		1.04 (1.01–1.07)		1.07 (1.02–1.11)		1.02 (0.97–1.07)		1.01 (0.96–1.07)
**Waist-to-hip ratio**											
<0.76	173	1 (Referent)	1 (Referent)	128	1 (Referent)	54	1 (Referent)	57	1 (Referent)	42	1 (Referent)
0.76–<0.79	198	1.06 (0.86–1.30)	1.07 (0.86–1.33)	135	0.98 (0.76–1.27)	70	1.22 (0.84–1.77)	52	0.84 (0.57–1.25)	60	1.36 (0.90–2.07)
0.79–<0.83	240	1.21 (1.00–1.48)	1.22 (0.99–1.50)	173	1.20 (0.95–1.53)	82	1.30 (0.90–1.87)	83	1.31 (0.92–1.86)	69	1.43 (0.94–2.16)
0.83–<0.88	232	1.13 (0.92–1.37)	1.17 (0.95–1.44)	175	1.15 (0.90–1.47)	97	1.42 (0.99–2.04)	74	1.12 (0.77–1.61)	60	1.39 (0.91–2.10)
≥0.88	277	1.31 (1.08–1.59)	1.33 (1.08–1.65)	200	1.29 (1.01–1.65)	114	1.80 (1.27–2.56)	78	1.06 (0.73–1.55)	70	1.50 (0.98–2.28)
*P*_*trend*_		*0.006*	*0.005*		*0.02*		<*0.0001*		*0.47*		*0.10*
Per 0.25 increase		1.41 (1.14–1.74)	1.49 (1.18–1.88)		1.54 (1.17–2.02)		1.93 (1.32–2.81)		1.36 (0.90–2.06)		1.46 (0.93–2.27)
**Total body fat %**											
<30.8	184	1 (Referent)	1 (Referent)	136	1 (Referent)	62	1 (Referent)	59	1 (Referent)	48	1 (Referent)
30.8–<35.0	223	1.09 (0.90–1.33)	1.05 (0.85–1.28)	151	0.95 (0.74–1.21)	81	1.12 (0.79–1.58)	62	0.90 (0.62–1.31)	70	1.34 (0.91–1.97)
35.0–<38.5	239	1.10 (0.91–1.34)	1.06 (0.86–1.29)	171	0.99 (0.79–1.27)	85	1.05 (0.74–1.48)	73	0.99 (0.69–1.42)	69	1.29 (0.87–1.91)
38.5–<42.5	237	1.04 (0.86–1.26)	1.01 (0.82–1.24)	176	1.01 (0.79–1.28)	99	1.21 (0.86–1.70)	72	0.93 (0.64–1.34)	57	0.99 (0.65–1.51)
≥42.5	227	1.04 (0.86–1.27)	0.99 (0.79–1.23)	173	1.00 (0.78–1.28)	88	1.05 (0.73–1.51)	76	1.05 (0.72–1.52)	52	1.02 (0.66–1.57)
*P*_*trend*_		*0.89*	*0.79*		*0.86*		*0.67*		*0.78*		*0.62*
Per 5% increase		0.99 (0.95–1.04)	0.99 (0.94–1.04)		1.00 (0.94–1.06)		1.03 (0.95–1.12)		0.98 (0.90–1.07)		0.96 (0.87–1.05)
**Trunk fat %**											
<27.7	183	1 (Referent)	1 (Referent)	134	1 (Referent)	62	1 (Referent)	56	1 (Referent)	49	1 (Referent)
27.7–<32.5	236	1.14 (0.94–1.39)	1.13 (0.92–1.38)	163	1.04 (0.82–1.33)	87	1.21 (0.87–1.70)	66	0.99 (0.68–1.43)	71	1.34 (0.91–1.98)
32.5–<36.4	229	1.08 (0.89–1.32)	1.01 (0.82–1.25)	171	1.02 (0.80–1.29)	81	0.98 (0.69–1.40)	80	1.14 (0.79–1.63)	59	1.07 (0.71–1.61)
36.4–<40.7	235	1.07 (0.88–1.30)	1.05 (0.86–1.30)	170	1.03 (0.81–1.31)	93	1.15 (0.82–1.62)	72	1.05 (0.73–1.52)	63	1.16 (0.77–1.75)
≥40.7	225	1.05 (0.86–1.27)	0.95 (0.76–1.18)	168	0.94 (0.73–1.21)	92	1.06 (0.74–1.52)	67	0.90 (0.61–1.33)	53	1.01 (0.66–1.55)
*P*_*trend*_		*0.92*	*0.50*		*0.63*		*0.87*		*0.73*		*0.80*
Per 5% increase		1.00 (0.96–1.04)	0.98 (0.94–1.03)		0.99 (0.94–1.05)		1.02 (0.94–1.09)		0.98 (0.90–1.06)		0.96 (0.88–1.04)

Basic model – Cox regression stratified by age (5-year categories), Townsend deprivation index fifths, and region of the recruitment assessment centre. Multivariable model – Cox regression using physical activity (MET hours/week; fifths), smoking status and intensity (never; former; current –<15 cigarettes/day; current –≥15 cigarettes/day; current – intensity unknown; unknown), alcohol consumption frequency (never; special occasions only; 1–3 times/month; 1–2 times/week; 3–4 times/week; daily or almost daily; unknown), family history of colorectal cancer (no; yes; unknown), prevalent diabetes (no; yes; unknown), regular aspirin/ibuprofen use (no; yes; unknown), qualifications (none; CSEs/O-levels/GCSEs or equivalent; NVQ/HND/HNC/A-levels/AS-levels or equivalent; other professional qualifications; college/university degree; unknown), ever use of hormone replacement therapy (no, yes, unknown), frequency of red and processed meat consumption (<2 times/week; 2–3 times/week; 3–4 times/week; ≥4 times/week; unknown), and stratified by age (5-year categories), Townsend deprivation index fifths, and region of the recruitment assessment centre.

Weight was associated with a higher risk of colorectal cancer among men (HR for highest versus lowest fifth = 1.57, 95%CI: 1.29–1.91; P_trend_ < 0.0001), but not women (HR for highest versus lowest fifth = 1.11, 95%CI: 0.89–1.38; P_trend_ = 0.56) (Tables 2 and 3). For men, this positive relationship was slightly stronger for colon cancer versus rectal cancer (P_heterogeneity_ = 0.09) and for proximal colon versus distal colon cancer (P_heterogeneity_ = 0.08), although these differences did not reach statistical significance. Among women, no differences in the weight and colorectal cancer relationship was found across anatomical subsites (colon versus rectal P_heterogeneity_ = 0.18; proximal colon versus distal colon P_heterogeneity_ = 0.12) (Table 3). Height was not associated with colorectal cancer risk among men (HR for highest versus lowest fifth = 1.01, 95%CI: 0.85–1.19; P_trend_ = 0.67) and women (HR for highest versus lowest fifth = 1.11, 95%CI: 0.90–1.37; P_trend_ = 0.30), with similar relationships found across anatomical subsites (all P_heterogeneities_ > 0.20) (Tables 2 and 3).

Among men, waist circumference and waist-to-hip ratio were associated with a greater risk of colorectal cancer (waist circumference: HR for highest versus lowest fifth = 1.66, 95%CI: 1.39–1.99; P_trend_ < 0.0001, waist-to-hip ratio: HR for highest versus lowest fifth = 1.58, 95%CI: 1.31–1.91; P_trend_ < 0.0001) (Table 2). Similar strength positive relationships were observed across anatomical subsites (colon versus rectal: waist circumference P_heterogeneity_ = 0.27, waist-to-hip ratio P_heterogeneity_ = 0.42; proximal colon versus distal colon: waist circumference P_heterogeneity_ = 0.27, waist-to-hip ratio P_heterogeneity_ = 0.61) (Table 2). For women, greater waist-to hip ratio was also associated with an elevated colorectal cancer risk (HR for highest versus lowest fifth = 1.33, 95%CI: 1.08–1.65; P_trend_ = 0.005) (Table 3). This positive relationship for waist-to-hip ratio was of similar magnitude for colon cancer and rectal cancer (P_heterogeneity_ = 0.84), and for proximal colon and distal colon cancer (P_heterogeneity_ = 0.24) (Table 3). Among women, waist circumference was positively related to colorectal cancer risk, although this relationship did not quite reach statistical significance (Table 3). Although no relationship was observed for waist circumference and rectal cancer in women, no heterogeneity was observed when compared with tumours in the colon (P_heterogeneity_ = 0.35). Waist circumference was positively associated with proximal colon cancer risk in women (HR for highest versus lowest fifth = 1.49, 95%CI: 1.04–2.14; P_trend_ = 0.04), but not distal colon cancer risk (P_heterogeneity_ = 0.15).

### Body fat composition and colorectal cancer risk

Among men, higher total body fat percentage and trunk fat percentage were associated with greater colorectal cancer risk (total body fat percentage: HR for highest versus lowest fifth = 1.27, 95%CI: 1.06–1.53; P_trend_ = 0.002, trunk fat percentage: HR for highest versus lowest fifth = 1.31, 95%CI: 1.09–1.58; P_trend_ = 0.002) (Table 2). These positive relationships were stronger and reached statistical significance for colon cancer, but not for rectal cancer (total body fat percentage P_heterogeneity_ = 0.21; trunk fat percentage P_heterogeneity_ = 0.24), and for proximal colon cancer, but not distal colon cancer (total body fat percentage P_heterogeneity_ = 0.90; trunk fat percentage P_heterogeneity_ = 0.64) (Table 2). For women, total body fat percentage and trunk fat percentage were not associated with colorectal cancer risk (Table 3).

### Sensitivity and sub-group analyses

The positive association observed between waist-to-hip ratio and colorectal cancer among women was evident among never users of HRT (HR per 0.25 increase = 1.42, 95%CI: 1.02–1.95) and ever users of HRT (HR per 0.25 increase = 1.60, 95%CI: 1.14–2.23) (P_interaction_ = 0.27) (Table 4). Similarly, the association of BMI and waist circumference with colorectal cancer did not differ across HRT use groups (P_interactions_ > 0.12). The associations between BMI, waist circumference, and waist-to-hip ratio with colorectal cancer risk were similar across subgroups of physical activity levels, prevalent diabetes status, and menopausal status (all P_interactions_ > 0.10) (Table 4). Similar relationships were also observed when colorectal cancer cases diagnosed during the first two years of follow-up were excluded from the analyses (data not shown).

**Table 4:** Subgroup analysis of the associations of body size measurements and colorectal cancer risk among men and women.

	**Body mass index, kg/m** ^**2**^		**Waist circumference, cm**		**Waist-to-hip ratio**	
	**HR (95% CI) per 2.5 kg/m** ^**2**^ **increment**	**HR (95% CI) per 2.5 kg/m** ^**2**^ **increment**	**HR (95% CI) per 5 cm increment**	**HR (95% CI) per 5 cm increment**	**HR (95% CI) per 0.25 increment**	**HR (95% CI) per 0.25 increment**
	**Men**	**Women**	**Men**	**Women**	**Men**	**Women**
**Physical activity levels** ^**†**^						
Low	1.08 (1.02–1.13)	0.98 (0.92–1.03)	1.07 (1.03–1.12)	1.02 (0.98–1.06)	1.62 (1.13–2.33)	1.49 (1.01–2.18)
Medium	1.05 (0.99–1.12)	1.03 (0.97–1.09)	1.06 (1.01–1.11)	1.03 (0.99–1.09)	2.02 (1.37–2.97)	1.54 (1.02–3.33)
High	1.08 (1.02–1.15)	1.04 (0.97–1.10)	1.07 (1.02–1.12)	1.05 (0.99–1.10)	1.90 (1.30–2.78)	1.45 (0.96–2.19)
P_interaction_	0.52	0.10	0.56	0.44	0.93	0.82
**Prevalent diabetes**						
No	1.08 (1.04–1.11)	1.01 (0.98–1.05)	1.06 (1.04–1.09)	1.03 (1.00–1.06)	1.78 (1.42–2.23)	1.46 (1.15–1.86)
Yes	1.04 (0.95–1.15)	0.99 (0.86–1.14)	1.09 (1.01–1.17)	1.03 (0.91–1.16)	2.21 (1.10–4.41)	2.29 (0.68–7.71)
P_interaction_	0.74	0.93	0.41	0.95	0.55	0.68
**Hormone replacement therapy use**						
Never use	—	1.00 (0.95–1.04)	—	1.02 (0.99–1.06)	—	1.42 (1.02–1.95)
Ever use	—	1.03 (0.98–1.08)	—	1.04 (1.00–1.08)	—	1.60 (1.14–2.23)
P_interaction_		0.23		0.12		0.27
**Menopausal status**						
Pre-menopausal	—	0.93 (0.84–1.03)	—	0.95 (0.88–1.04)	—	1.15 (0.57–2.35)
Post-menopausal	—	1.01 (0.97–1.05)	—	1.04 (1.00–1.07)	—	1.58 (1.20–2.06)
P_interaction_		0.90		0.66		0.50

All HRs per specified unit increase. Multivariable models only – Cox regression using physical activity (MET hours/week; fifths), smoking status and intensity (never; former; current -<15 cigarettes/day; current -≥15 cigarettes/day; current – intensity unknown; unknown), alcohol consumption frequency (never; special occasions only; 1–3 times/month; 1–2 times/week; 3–4 times/week; daily or almost daily; unknown), family history of colorectal cancer (no; yes; unknown), prevalent diabetes (no; yes; unknown), regular aspirin/ibuprofen use (no; yes; unknown), qualifications (none; CSEs/O-levels/GCSEs or equivalent; NVQ/HND/HNC/A-levels/AS-levels or equivalent; other professional qualifications; college/university degree; unknown), ever use of hormone replacement therapy (no, yes, unknown), frequency of red and processed meat consumption (<2 times/week; 2–3 times/week; 3–4 times/week;≥4 times/week; unknown), and stratified by age (5-year categories), Townsend deprivation index fifths, and region of the recruitment assessment centre. ^**†**^Physical activity (MET hours/week) thirds used to categorise participants into low (lower third), medium (middle-third), and high (upper-third) levels of activity.

## Discussion

In this prospective analysis of UK Biobank participants, overall and abdominal adiposity body size and composition measurements were positively related to colorectal cancer risk among men. For women, only greater abdominal adiposity was associated with elevated colorectal cancer risk, and this relationship did not differ by HRT use.

Our finding that BMI was positively related to colorectal cancer risk for men is in accordance with a large body of epidemiological evidence^1,12^. For women, we observed no association between BMI and colorectal cancer development, which is inconsistent with a recent analysis from the Million Women’s Study (n = 18,518 cases)^13^ and two meta-analyses of >30 individual prospective studies^1,12^. This suggests that the null finding we observed between BMI and colorectal cancer among women may be the consequence of insufficient follow-up time and recorded case numbers that are necessary to detect the weak-to-moderate strength association which has previously been reported.

We found that greater abdominal adiposity measurements were positively related to colorectal cancer risk in men and women, findings consistent with the EPIC study and meta-analyses of prospective studies^1,4^, but inconsistent with the findings from a National Institutes of Health (NIH)-AARP Diet and Health Study analysis that reported positive associations for waist circumference and waist-to-hip ratio for men, but null relationships for women^6^.

This was the first large-scale study to investigate how body fat composition measurements relate to colorectal cancer risk. Analyses of total body fat percentage and colorectal cancer revealed similar relationships to those observed for BMI. For men, greater total body fat percentage was associated with elevated risks of developing colorectal cancer and colon cancer, a finding in that is in accordance with results from a Swedish cohort study which reported a RR of 1.54 (95%CI: 1.06–2.23; P-trend = 0.012) for colorectal cancer (n = 584 cases) when the highest and lowest fourths were compared^14^. For women, we observed a null relationship between colorectal cancer risk and total body fat percentage, a result consistent with a sub-study of 11,124 post-menopausal women (n = 169 cases) from the Women’s Health Initiative^15^. For trunk fat percentage, we found a positive relationship with colorectal cancer and colon cancer risk for men, and null associations for women. In general, our results for body fat composition were consistent with our findings for body size measurements.

It is not known why the positive relationships between adiposity and colorectal cancer are weaker and more inconsistent for women. One proposed explanation, is that greater exposure to estrogens in women may mitigate the potential tumorigenic effects of excess adiposity on the colorectum^3^. Experimental and epidemiological studies suggest that estrogens are potentially protective against the development of colorectal cancer^16–20^, while in observational studies, ever use of HRT has been consistently associated with lower colorectal cancer risk^2^. In the EPIC study, abdominal adiposity was only associated with greater colon cancer risk among non-HRT users, with no relationship observed for HRT users^4^. However, in the current UK Biobank study, we found no evidence that HRT use modified the abdominal adiposity and colorectal cancer relationship, as we observed consistent positive relationships among users and never users of HRT. There is evidence that the different formulations of HRT (e.g. estrogen only or estrogen plus progestin) may have distinct associations with colorectal cancer risk^21,22^. We did not have information on the formulation of HRT used which may explain why we did not observe effect modification of the abdominal adiposity and colorectal cancer relationship by HRT use.

Nonetheless, it remains possible that higher endogenous circulating levels of estrogens found in women may lessen the impact of adiposity on colorectal cancer development. Most recently, a case-control analysis nested within the Women’s Health Initiative found that higher levels of endogenous circulating estradiol and estrone were associated with a lower colorectal cancer risk in post-menopausal women who were non-HRT users^23^. In the same study, the positive waist circumference and colorectal cancer relationship was strengthened, and became statistically significant, after the multivariable models were additionally adjusted for endogenous circulating estrogen concentrations^23^. This suggests that higher endogenous circulating estrogen concentrations may mask the body size and colorectal cancer relationship in post-menopausal women, and that future studies investigating how adiposity relates to colorectal cancer development in post-menopausal women should incorporate estrogen measurements to limit the effects of this confounding bias^23^.

The biological mechanisms that underlie the relationship between adiposity and colorectal cancer development are uncertain. Adiposity is associated with metabolic and endocrinologic abnormalities, such as alterations in insulin and insulin-like growth factor signalling. Experimental and epidemiological evidence indicate that hyperinsulinemia and elevated levels of insulin-like growth factor- 1 (IGF-1) may promote colorectal tumorigenesis by stimulating mitogenesis and inhibiting apoptosis^24–30^. Excess body weight is considered a chronic inflammatory state characterised by increased adipose secretion of proinflammatory cytokines, such as interleukin-6 (IL-6), which has been shown to promote tumour initiation in experimental models^31^. Higher adiposity is also inversely related to the secretion of adiponectin, an adipose tissue secreted adipokine which has been shown to lower secretion of inflammatory cytokines, improve insulin sensitivity, and inhibit cell growth and angiogenesis^32,33^. In epidemiological studies, higher adiponectin levels have, in general, been associated with lower colorectal cancer risk^34,35^.

The current analysis represents one of the largest single studies investigating the relationships between body size and composition with colorectal cancer risk to date. This was the first time that bioelectrical impedance measurements, that distinguish between adipose and non-adipose mass, have been used on such a large-scale to assess how total body fat percentage and trunk fat percentage relate to colorectal cancer development. The large number of incident colorectal cancer cases allowed analyses by HRT use, physical activity levels, diabetes status, menopausal status, and tumour anatomical site, and the detailed phenotypic information collected from UK Biobank participants enabled us to carefully adjust for known colorectal cancer risk factors. A limitation of our analysis is that body size and composition measurements were collected from all participants on one occasion; however, with an average follow-up of 5.6 years it is unlikely that body measurements would have changed markedly during this time to substantially alter our results.

In conclusion, in this prospective analysis of UK Biobank participants, which used the most comprehensive panel of anthropometric measurements of any study to date, greater body size (overall and abdominal adiposity) was positively associated with colorectal cancer development in men. For women, abdominal adiposity, rather than overall body size, was associated with a greater colorectal cancer risk, and this relationship was unaffected by HRT use. The null result observed for the BMI and colorectal cancer among women may have been a consequence of insufficient follow-up time and recorded case numbers to detect the weak-to-moderate strength association which has previously been reported. Our findings add to the large body of evidence which supports the promotion of weight control in population-wide cancer prevention programmes.
